# Learning Curve and Clinical Outcome of Biportal Endoscopic-Assisted Lumbar Interbody Fusion

**DOI:** 10.1155/2020/8815432

**Published:** 2020-12-17

**Authors:** Ju-Eun Kim, Hyun-Seung Yoo, Dae-Jung Choi, Jin-Ho Hwang, Eugene J. Park, Seungho Chung

**Affiliations:** ^1^Department of Orthopedic Surgery, Himnaera Hospital, Busan, Republic of Korea; ^2^Department of Orthopedic Surgery, Daegu Fatima Hospital, Daegu, Republic of Korea; ^3^Department of Orthopedic Surgery, Kyungpook National University Hospital, Kyungpook National University School of Medicine, Daegu, Republic of Korea

## Abstract

Interbody fusion is a common surgical technique for diseases of the lumbar spine. Biportal endoscopic-assisted lumbar interbody fusion (BE-LIF) is a novel minimally invasive technique that has a long learning curve, which can be a barrier for surgeons. Therefore, we analyzed the learning curve in terms of operative time and evaluated the outcomes of BE-LIF. A retrospective study of fifty-seven consecutive patients who underwent BE-LIF for degenerative lumbar disease by a single surgeon from January 2017 to December 2018 was performed. Fifty patients underwent a single-level procedure, and 7 underwent surgery at two levels. The mean follow-up period was 24 months (range, 14–38). Total operative time, postoperative drainage volume, time to ambulation, and complications were analyzed. Clinical outcome was measured using the Oswestry Disability Index (ODI), Visual Analog Scale (VAS) score for back and leg pain, and modified Macnab criteria. The learning curve was evaluated by a nonparametric regression locally weighted scatterplot smoothing curve. Cases before the stable point on the curve were designated as group A, and those after the stable point were designated group B. Operative time decreased as the number of cases increased. A stable point was noticed on the 400th day and the 34th case after the first BE-LIF was performed. All cases showed improved ODI and VAS scores at the final follow-up. Overall mean operative time was 171.74 ± 35.1 min. Mean operative time was significantly lower in group B (139.7 ± 11.6 min) compared to group A (193.4 ± 28.3 min). Time to ambulation was significantly lower in group B compared to group A. VAS and ODI scores did not differ between the two groups. BE-LIF is an effective minimally invasive technique for lumbar degenerative disease. In our case series, this technique required approximately 34 cases to reach an adequate performance level.

## 1. Introduction

Harms first introduced the traditional open transforaminal lumbar interbody fusion (TLIF) in 1982 [1]. Although the technique reduces the need for thecal sac and nerve root retraction during cage insertion, it is associated with significant postoperative soft tissue injury [2–4]. Thus, minimally invasive transforaminal lumbar interbody fusion (MI-TLIF) was introduced. According to numerous studies, MI-TLIF has similar long-term clinical outcomes and fusion rates with less postoperative pain and shorter length of stay compared to open TLIF [5–7]. Though MI-TLIF requires less muscle dissection and causes less local tissue injury than open TLIF, both techniques require the use of retractors that cause muscle injury. Retractor pressure on the erector spinae muscles is related to postoperative spinal muscle dysfunction and operative scar generation [3, 8]. In addition, muscle retraction time > 60 minutes significantly affects clinical outcome measures for back pain, such as the Visual Analog Scale (VAS) score, Oswestry Disability Index (ODI), and the short form (SF)-36 questionnaire score [9].

Among the recently developed minimally invasive spinal surgical techniques, biportal endoscopic spinal surgery (BESS) has been applied for decompression as well as TLIF. Biportal endoscopic-assisted lumbar interbody fusion (BE-LIF) utilizes two separate percutaneous portals (viewing and working). It has several advantages over MI-TLIF due to its percutaneous approach, including minimization of retractor-induced muscle ischemia [10, 11]. Although the technical details and clinical outcomes of BE-LIF have been reported, there have been no studies of its learning curve. Therefore, we aimed to examine operative time to characterize the BE-LIF learning curve and evaluate its complications and clinical and radiographic outcomes. In addition, we compared the clinical and radiographic outcomes between the early and later cases.

## 2. Materials and Methods

We performed a retrospective study by reviewing the electronic medical records. This study was conducted after obtaining approval from the Institutional Review Board. Fifty-seven consecutive patients that underwent BE-LIF for lumbar degenerative disease by a single surgeon from January 2017 to December 2018 were enrolled. The indications of lumbar degenerative diseases requiring BE-LIF were degenerative spondylolisthesis and isthmic spondylolisthesis. We did not perform BE-LIF for revision or trauma cases. All patients received a minimum one-year follow-up, with the mean follow-up period of 24 months (range, 14–38).

### 2.1. Surgical Technique

**Figure 1:**
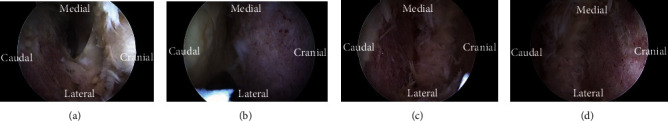
Intraoperative endoscopic views of the disc space. (a) Double-ended elevator used to denude the superior endplate of the caudal vertebra. (b) Denuded inferior endplate of the cranial vertebra. (c) Denuded superior and inferior endplates of the disc space (caudally tilted view). (d) Denuded superior and inferior endplate of the disc space (cranially tilted view).

**Figure 2:**
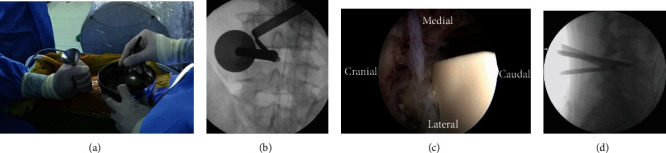
Bone graft and cage insertion. (a) A funnel is used to insert bone graft inside the disc space. (b) Posteroanterior fluoroscopic view of the funnel positioned inside the disc space. (c) Cage inserted under endoscope guidance. (d) Lateral fluoroscopic view during the cage insertion.

The patient was positioned prone on a radiolucent operating table under general anesthesia. An arthroscopic imaging system for joint surgery was used for all cases. Initially, a true anteroposterior fluoroscopic view of the surgical site was obtained. For a left-sided approach, the proximal viewing portal and distal working portal were placed 1 cm above and below the surgical level, respectively. Both portal sites were used later as the entry points for percutaneous pedicle screws. For each portal, a 1 cm transverse skin incision was made; the fascial incision was made perpendicular to the skin incision to allow fluent outflow of irrigation fluid, which is essential for a clear intraoperative visual field. With fluoroscopic guidance, Cobb elevators were inserted percutaneously through the two portals. They were used to dissect the muscle away from the interlaminar space and proximal lamina to create a working space. Gravity-powered irrigation was used from a saline bag suspended at the height of 1.7 meters from the floor. To prevent obstruction of irrigation fluid outflow, a semitubular retractor was manually placed and held through the working portal. Soft tissue debridement and hemostasis were achieved using a radiofrequency coagulator. The subsequent steps resemble the MI-TLIF procedure. Proximal laminotomy and unilateral facetectomy were performed using a high-speed burr, Kerrison rongeur, and osteotome. Local autologous bone obtained during decompression was set aside for later use as bone graft. After the laminotomy and facetectomy, bilateral flavectomy was performed, and the shoulder portion of the traversing root and the disc space was exposed. Then, annulotomy and disc removal were performed. Tapered trial cages were serially inserted to expand the disc space adequately to allow insertion of the endoscope and instruments into the disc space. The endplates were prepared by gently removing the cartilage using a double-ended elevator while avoiding subchondral bone damage (Figure 1). The local autologous bone was then packed into the anterior aspect of the disc space using a funnel, and the cage was carefully inserted under endoscopic guidance to avoid injury of the exiting and traversing nerve roots (Figure 2). Finally, percutaneous pedicle screws were inserted bilaterally. Unilateral percutaneous pedicle screws were inserted through the two endoscopic portals, and the contralateral unilateral percutaneous pedicle screws were inserted with conventional skin incisions.

### 2.2. Clinical and Radiological Outcome Measures

**Figure 3:**
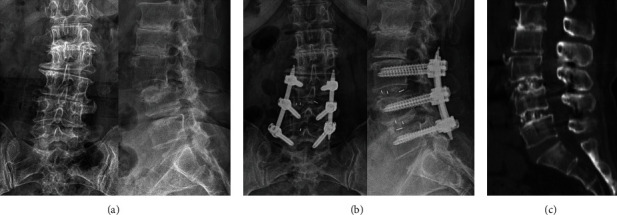
Perioperative plain radiography films. (a) Preoperative anteroposterior and lateral images show degenerative L3–4 spondylolisthesis. (b) Follow-up anteroposterior and lateral films showing reduction of sagittal alignment. (c) Sagittal computed tomography image 14 months postoperatively showing trabecular bridging without endplate disruption or cage subsidence.

Operative time, time to ambulation, length of stay, postoperative drainage amount, and complications were analyzed. The group before the stable point in terms of operative time was designated group A, and the group after the stable point was designated group B. A stable point was defined as the point that the line afterwards shows the smoothest trend. VAS and ODI data from before surgery and two weeks, two months, and after 1 year postoperatively were collected to evaluate lower back pain, leg pain, and disability. The modified Macnab criteria were checked on the final follow-up visit of each patient. For preoperative radiographic evaluation, anteroposterior, lateral, oblique, and flexion-extension lumbar plain radiographs, CT, and MRI were used. Interbody fusion was evaluated one year after surgery using lateral and flexion-extension plain radiographs. Radiographic fusion was assessed by two board-certified radiologists and defined as either formation of a trabecular bone bridge or <4 degrees of segmental motion on flexion-extension plain radiographs. The Bridwell grading system was used to classify fusion status [12] (Figure 3).

### 2.3. Statistical Analysis

Data are reported as means ± standard deviation. A nonparametric regression locally weighted scatterplot smoothing curve was used to analyze the change of operative time, with the *x*-axis showing the date after the first case and the *y*-axis showing operative time. The chi-square test or two-sample *t*-test was used to compare clinical characteristics between the two groups. Clinical and radiographic results were compared using the two-sample *t*-test, chi-square test, Fisher's exact test, or repeated-measures ANOVA. SPSS software version 24.0 (IBM, Armonk, NY, USA) and MedCalc software version 19.1 were used for statistical analysis. *p* < 0.05 was considered significant.

## 3. Results

### 3.1. Demographic Data

The mean patient age was 68.5 ± 9.4 years. Twenty-eight patients were male. Patient diagnoses included 46 cases of degenerative spondylolisthesis and 11 cases of isthmic spondylolisthesis. Fifty patients underwent single-level TLIF, and 7 underwent 2-level TLIF. The level of surgery in the single-level cases was L2-3 in 1, L3-4 in 5, L4-5 in 26, and L5-S1 in 18. Two-level TLIF was performed at L3-4 and L4-5 in 5 patients and L4-5 and L5-S1 in 2. Patient characteristics are summarized in Table 1. There was no significant difference between the two groups in all data variables.

### 3.2. Learning Curve and Clinical Outcome

**Figure 4:**
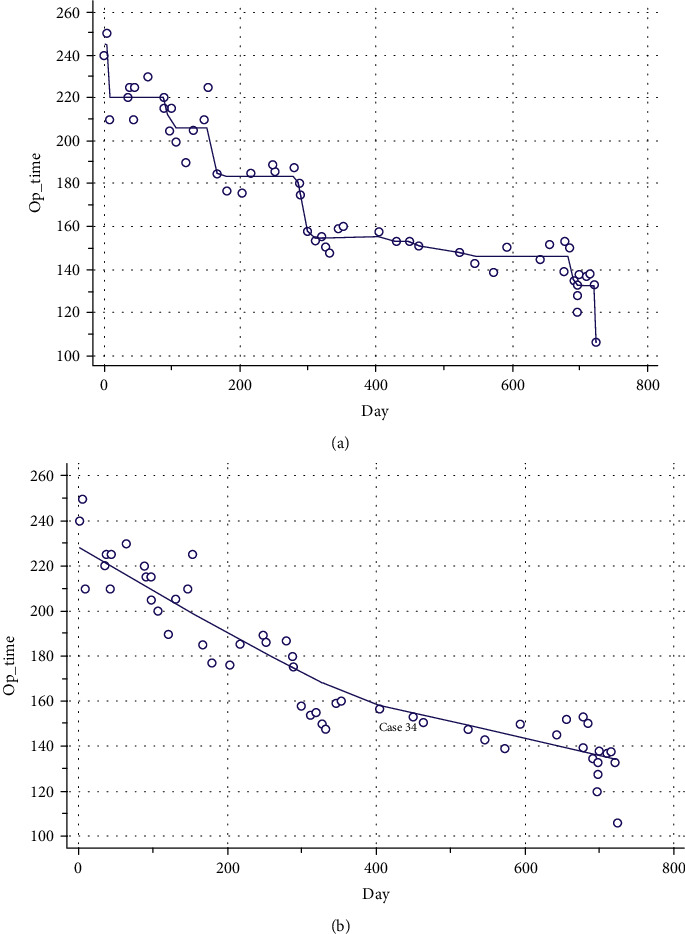
A nonparametric regression locally weighted scatterplot smoothing curve of operative time and day after the first biportal endoscopic-lumbar interbody fusion case (a, b). The slope of operative time flattened after the 34th case and 400th day.

**Figure 5:**
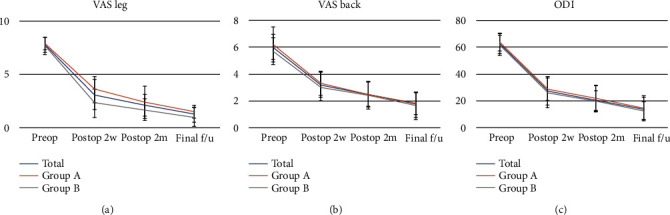
Pain and functional scores. The values between the 2 groups at each time point did not show a significant difference. (a) VAS leg score significantly improved after surgery. (b) VAS back score significantly improved after surgery. (c) ODI score significantly improved after surgery (VAS: Visual Analog Scale; ODI: Oswestry Disability Index).

The operative time decreased as the number of cases increased. Using a nonparametric regression locally weighted scatterplot smoothing curve, the slope of operative time flattened after the 34th case and 400th day after the 1st case of BE-LIF (Figure 4). The mean operative time was 171.7 ± 35.1 minutes. The operative time in group B (139.7 ± 11.6 min) was significantly shorter than group A (193.4 ± 28.3 min) (*p* ≤ 0.001) (Table 1). The ODI, VAS leg, and VAS back scores significantly improved after surgery throughout the follow-up period in both groups (Figure 5). Unlike the ODI and VAS back scores, the change in VAS leg pain change significantly differed between the two groups (*p* = 0.007) (Table 2). While the VAS leg score improvement at two months postoperatively and final follow-up did not reach significance, the improvement at two weeks was significantly lower in group A (Table 3). Modified Macnab criteria, Bridwell grade, complications, revision rate, and length of stay were not different between the two groups. Time to ambulation was significantly longer in group A (13.7 ± 4.0 h) compared to group B (9.9 ± 3.7 h) (Table 4).

### 3.3. Complications

A complication occurred in 3 of the 57 patients (5.3%): 1 postoperative spinal epidural hematoma, 1 case of cage subsidence, and 1 case of transient paralysis. The postoperative spinal epidural hematoma and transient paralysis occurred in the 17^th^ and 24^th^ case, respectively, of group A and cage subsidence was found in group B. The postoperative spinal epidural hematoma was managed with revision surgery of surgical hematoma evacuation, which resulted in the improvement of back pain and neurologic symptoms.

## 4. Discussion

Minimally invasive spinal surgery is effective in reducing soft tissue damage and recovery time compared to open surgery [13]. Numerous minimally invasive techniques for addressing lumbar degenerative diseases have been introduced. Aided by recent advances in optics, endoscopic techniques for discectomy and decompression through a posterior approach have shown promising outcomes [10, 11, 14–16]. Techniques for posterolateral fusion, lateral interbody fusion, and MI-TLIF using tubular retractors have been reported. MI-TLIF is an advanced technique of traditional TLIF that minimizes nerve root traction [17]. MI-TLIF preserves the contralateral paraspinal muscles and facets [18] and reduces the length of hospital stay and postoperative pain, which leads to earlier recovery [13]. However, minimally invasive techniques have a long learning curve, which is a barrier for surgeons familiar with open surgery [19, 20]. MI-TLIF is performed within a relatively small visual field through a tubular retractor; thus, contralateral decompression can be difficult, depending on the surgeon's experience and anatomy of the patient. Although a minimally invasive technique, MI-TLIF, was still associated with muscle ischemia due to tissue pressure from the tubular retractor, correlation of postoperative back muscle performance and muscle retraction time has been previously reported [8]. Muscle retraction time > 60 minutes is related to worse pain and function scores [21].

Theoretically, since BE-LIF is performed percutaneously without retractors, muscle damage is presumably less compared to MI-TLIF. A previous study comparing BESS and microscopic surgery for unilateral laminotomy and bilateral decompression showed favorable results for BESS in terms of early postoperative clinical outcome. The authors assumed that such results were due to less tissue damage during BESS than open microscopic surgery [14]. However, BE-LIF is technically demanding especially for surgeons without endoscopic experience and is associated with a long learning curve. Since the first introduction of BE-LIF, only preliminary results have been reported without data regarding the learning curve or clinical and radiographic outcomes [10, 11]. Park et al. reported that the learning curve for lumbar decompression using BESS in trainees without endoscopic experience reached a stable operative time after 58 cases [22]. Few studies regarding the learning curve for MI-TLIF have been reported [19, 23]. According to Lee et al., MI-TLIF performed through a unilateral approach reduces the operative time and soft tissue damage compared to posterior lumbar interbody fusion; a plateau in operative time was achieved after a certain time and performing a certain number of cases [24]. In our study, the learning curve for BE-LIF showed a gradual reduction in operative time until the 34th case, which we consider a stable point. Although the mean operative time of the entire cohort was 171.7 ± 35.1 min, the mean operative time for cases before the stable point was 193.4 ± 28.3 min, which was significantly longer than the cases after the stable point (139.7 ± 11.6 min). The operation time was prolonged in earlier cases due to discectomy and endplate preparation. As the surgeon became more familiar with the triangulation and manipulation of the instruments removing discs and denuding the endplates, the operative time decreased subsequently. Another study of the MI-TLIF learning curve showed that 30 cases are required for technical proficiency in terms of operative time; the mean operative time of the first 30 cases was 254 ± 44 min, while the mean time afterward significantly decreased to 183 ± 23 min [19]. Similar to our study, other studies have shown that MI-TLIF operative times range between 150 and 220 min [5, 20, 25].

Previously reported MI-TLIF complication rates were zero to 33% [19, 23]. In our study, the BE-LIF complication rate was 5.2% and did not differ between early and late cases. VAS and ODI scores significantly improved over time in both groups. However, the later cases showed better early postoperative results in VAS leg pain score and time to ambulation, which is likely related to shorter operative and anesthesia time. Also, the complication of transient paralysis only occurred in group A. The possible mechanism of such results is assumed to be prolonged operative time and the “battered” root problem [26]. In the early cases, we may have excessively retracted and manipulated the dura and nerve root to avoid nerve roots being entangled during cage insertion. However, after cases, we have become more familiar with the corridor for cage insertion, which was wide enough that nerve roots retraction was unnecessary. Lee et al. similarly analyzed the learning curve for MI-TLIF and concluded that there was no significant difference between early and later cases in terms of final outcome except for time to ambulation. However, they did not compare early postoperative results [19].

In our case series, 55 of the 57 cases showed Bridwell grade I or II interbody fusion. Although the follow-up period was longer than the whole cohort, 2 cases showed grade III fusion in the early group. MI-TLIF has been reported to result in fusion rates ranging from 80% to 100% [5, 19, 20, 27], whereas BE-LIF reports note fusion rates of at least 78% [25, 28]. The first BE-LIF study showed interbody fusion in 18 of 23 cases (78%); the authors noted that the relatively short 1-year follow-up period might explain the low fusion rate. Park et al. analyzed 61 BE-LIF cases and found that fusion was achieved in 58 cases (95%): Bridwell grade I in 43 and Bridwell grade II in 15 [25]. These results are nearly identical to the 96% interbody fusion rate found in our study. Numerous studies have mentioned that one advantage of BE-LIF is endplate preparation under direct and clear visualization (.1) [11, 25, 28]. Meticulous endplate preparation with minimal subchondral bone injury provides an adequate fusion bed and enhances the fusion rate. However, endplate preparation and cage insertion with other lumbar interbody fusion techniques are performed blindly, which may result in inadequate preparation of the fusion bed.

However, BE-LIF has potential complications during surgery. For surgeons with insufficient experience, inadequate hemostasis, loss of orientation can prolong the operative time and may result in minor complications such as postoperative epidural hematoma, iatrogenic durotomy, or injury to the nerve root to major complications including nerve root palsy or intraspinal hematoma. Therefore, endoscopic training from cadaveric laboratories is recommended before attempting such technique [29, 30].

There are several limitations to this study. First, this is a retrospective study investigating a heterogeneous disease entity. Second, only the duration of the entire operation rather than the individual operation steps such as laminotomy, discectomy, endplate preparation, or cage insertion was recorded. Third, the sample size is relatively small with a short follow-up period. Finally, there is a performance bias in this study that the operating surgeon has been performing BESS for decompression or discectomy for the last 1 year before starting BE-LIF. The required cases for adequate performance cannot be generalized since the actual learning curve can vary among individuals.

## 5. Conclusions

BE-LIF is an effective minimally invasive technique for lumbar degenerative disease. It can result in significant clinical improvement with a low risk of complications, which seems to be comparable to the results of MI-TLIF. In our case series, 34 cases were required to reach a constant and adequate performance level.

## Figures and Tables

**Table 1:** Patient characteristics. Statistical analysis between the two groups by chi-square test or two sample *t*-test (VAS: Visual Analog Scale; ODI: Oswestry Disability Index; ^∗^*p* value < 0.05).

Variables		Total (*n* = 57)	Group A (*n* = 34)	Group B (*n* = 23)	*p* value
Operative time (min)^∗^		171.74 ± 35.1	193.4 ± 28.3	139.7 ± 11.6	≤0.001
Age (year)		68.5 ± 9.4	69.8 ± 8.6	66.7 ± 10.2	0.227

Sex	M	28 (50%)	18 (32%)	10 (18%)	0.592
	F	29 (50%)	16 (28%)	13 (23%)	

Operation extent (levels)	1	49 (86%)	32 (56%)	17 (30%)	0.069
	2	8 (15%)	2 (4%)	6 (11%)	

Index level	L2-3	1 (2%)	1 (2%)	0 (0%)	0.080
	L3-4	10 (18%)	4 (7%)	6 (11%)	
	L4-5	28 (49%)	21 (37%)	7 (12%)	
	L5-S1	18 (32%)	8 (14)	10 (18%)	

**Table 2:** Comparison of clinical outcome scores divided by group. Statistical analysis between the two groups by repeated measures ANOVA (VAS: Visual Analog Scale; ODI: Oswestry Disability Index; ^∗^*p* value < 0.05).

Variables		Total	Group A	Group B	*p* value
VAS leg^∗^	Preop	7.8 ± 0.7	7.9 ± 0.6	7.7 ± 0.8	0.007
	Postop 2 wks	3.1 ± 1.4	3.6 ± 1.2	2.3 ± 1.3	
	Postop 2 mo	2.1 ± 1.0	2.4 ± 1.0	1.7 ± 1.0	
	Final	1.3 ± 0.8	1.5 ± 0.6	1.0 ± 0.9	

VAS back	Preop	6.0 ± 1.1	6.2 ± 1.3	5.7 ± 0.9	0.637
	Postop 2 wks	3.2 ± 0.9	3.3 ± 0.9	3.0 ± 0.9	
	Postop 2 mo	2.5 ± 0.9	2.5 ± 0.9	2.4 ± 0.9	
	Final	1.7 ± 0.8	1.8 ± 0.8	1.4 ± 0.6	

ODI	Preop	65.0 ± 7.6	63.6 ± 5.8	62.6 ± 8.4	0.358
	Postop 2 wks	29.2 ± 10.6	28.9 ± 8.4	26.3 ± 10.8	
	Postop 2 mo	22.2 ± 9.7	22.6 ± 9.3	20.4 ± 7.4	
	Final	16.1 ± 8.9	14.9 ± 9.0	13.0 ± 6.6	

**Table 3:** Comparison of improvement in clinical outcome scores divided by group. Statistical analysis by two sample *t*-test (VAS: Visual Analog Scale; ODI: Oswestry Disability Index; pre: preoperative; PO: postoperative; w: weeks; m: months, ^∗^*p* value < 0.05).

Variables		Group A	Group B	*p* value
VAS leg	Pre-PO 2 w^∗^	4.3 ± 1.1	5.4 ± 1.2	0.001
	Pre-PO 2 m	5.6 ± 1.0	6.0 ± 0.9	0.122
	Pre-final	6.5 ± 0.9	6.7 ± 0.9	0.255

VAS back	Pre-PO 2 w	2.9 ± 1.4	2.7 ± 0.9	0.637
	Pre-PO 2 m	3.7 ± 1.4	3.4 ± 0.9	0.349
	Pre-final	4.4 ± 1.3	4.3 ± 1.0	0.726

ODI	Pre-PO 2 w	34.7 ± 7.6	36.3 ± 8.0	0.131
	Pre-PO 2 m	41.1 ± 8.0	42.2 ± 5.0	0.141
	Pre-final	48.7 ± 7.0	49.6 ± 4.6	0.431

**Table 4:** Other clinical outcome measures divided by group. Statistical analysis between the two groups by chi-square test, fisher's exact test, or two sample *t*-test (^∗^*p* value < 0.05).

Variables		Total	Group A	Group B	*p* value
Modified Macnab criteria	1	32 (56.1%)	15 (26.3%)	17 (29.8%)	0.143
	2	18 (31.6%)	14 (24.6%)	4 (7.0%)	
	3	6 (10.5%)	4 (7.0%)	2 (3.5%)	
	4	1 (1.8%)	1 (1.8%)	0 (0.0%)	

Complication	Y	3 (5.3%)	2 (3.5%)	1 (1.8%)	0.799
	N	54 (94.7%)	32 (56.1%)	22 (38.6%)	

Bridwell grade	1	41 (71.9%)	21 (36.8%)	20 (35.1%)	0.098
	2	14 (24.6%)	11 (19.3%)	3 (5.3%)	
	3	2 (3.5%)	2 (3.5%)	0 (0.0%)	

Revision	Y	2 (3.5%)	1 (1.8%)	1 (1.8%)	0.777
Postoperative drain amount (ml)		113.9 ± 38.4	117.1 ± 36.9	109.1 ± 41.0	0.460
Time to ambulation (hours)^∗^		12.2 ± 4.3	13.7 ± 4.0	9.9 ± 3.7	≤0.001
Length of stay (days)		7.1 ± 3.3	7.0 ± 3.0	7.4 ± 3.9	0.696

## Data Availability

All equipment and materials used in this work are described, and all relevant results obtained were presented and discussed (tables). Other details can be requested.
